# Evolution of the *EKA* family of powdery mildew avirulence-effector genes from the ORF 1 of a LINE retrotransposon

**DOI:** 10.1186/s12864-015-2185-x

**Published:** 2015-11-10

**Authors:** Joelle Amselem, Marielle Vigouroux, Simone Oberhaensli, James K. M. Brown, Laurence V. Bindschedler, Pari Skamnioti, Thomas Wicker, Pietro D. Spanu, Hadi Quesneville, Soledad Sacristán

**Affiliations:** INRA, UR1164 URGI Unité de Recherche Génomique-Info, Institut National de la Recherche Agronomique de Versailles-Grignon, Versailles, 78026 France; INRA, UR1290 BIOGER, Biologie et gestion des risques en agriculture, Campus AgroParisTech, 78850 Thiverval-Grignon, France; John Innes Centre, Norwich Research Park, Norwich, NR4 7UH UK; Institute of Plant Biology, University of Zurich, Zollikerstrasse 107, 8008 Zurich, Switzerland; School of Biological Sciences, Royal Holloway University of London, Egham, TW20 0EX UK; Laboratory of Genetics, Department of Biotechnology, Agricultural University of Athens, Iera Odos 75, TK 11855 Athens, Greece; Department of Life Sciences, Imperial College London, London, UK; Centro de Biotecnología y Genómica de Plantas (UPM-INIA) and E.T.S.I. Agrónomos, Universidad Politécnica de Madrid, Campus de Montegancedo, Pozuelo de Alarcón, 28223 Madrid Spain

**Keywords:** Barley, Powdery mildew, *Blumeria graminis*, Effector, Retrotransposon, LINE, Co-option

## Abstract

**Background:**

The *Avrk1* and *Avra10* avirulence (AVR) genes encode effectors that increase the pathogenicity of the fungus *Blumeria graminis* f.sp. *hordei* (*Bgh*), the powdery mildew pathogen, in susceptible barley plants. In resistant barley, MLK1 and MLA10 resistance proteins recognize the presence of AVRK1 and AVRA10, eliciting the hypersensitive response typical of gene for gene interactions. *Avrk1* and *Avra10* have more than 1350 homologues in *Bgh* genome, forming the *EKA* (Effectors homologous to *Avrk1* and *Avra10)* gene family.

**Results:**

We tested the hypothesis that the *EKA* family originated from degenerate copies of Class I LINE retrotransposons by analysing the *EKA* family in the genome of *Bgh* isolate DH14 with bioinformatic tools specially developed for the analysis of Transposable Elements (TE) in genomes. The Class I LINE retrotransposon copies homologous to *Avrk1* and *Avra10* represent 6.5 % of the *Bgh* annotated genome and, among them, we identified 293 AVR/effector candidate genes. We also experimentally identified peptides that indicated the translation of several predicted proteins from *EKA* family members, which had higher relative abundance in haustoria than in hyphae.

**Conclusions:**

Our analyses indicate that *Avrk1* and *Avra10* have evolved from part of the ORF1 gene of Class I LINE retrotransposons*.* The co-option of *Avra10* and *Avrk1* as effectors from truncated copies of retrotransposons explains the huge number of homologues in *Bgh* genome that could act as dynamic reservoirs from which new effector genes may evolve. These data provide further evidence for recruitment of retrotransposons in the evolution of new biological functions.

**Electronic supplementary material:**

The online version of this article (doi:10.1186/s12864-015-2185-x) contains supplementary material, which is available to authorized users.

## Background

Plant pathogens secrete effectors to influence host metabolism or defense mechanisms to provide an environment for successful infection [1]. Effectors can be recognized by plant resistance (R) genes, eliciting effector-triggered immunity (ETI) in the plant to prevent further infection [2]. Effectors that are recognized by the plant are known as avirulence (AVR) proteins in the context of gene-for-gene interactions. Alteration of AVR genes enables parasites to avoid R-dependent recognition and thus overcome host resistance [3].

The *Avrk1* and *Avra10* genes of the barley powdery mildew fungus, *Blumeria graminis* f.sp. *hordei* (*Bgh*), encode proteins which have a dual role as effectors and as AVR proteins, the presence of which is recognized by the MLK1 and MLA10 R-proteins. These genes were cloned from isolate CC148 (avirulent to barley plants carrying *Mlk1* or *Mla10R*- genes) by genetic and physical mapping [4]. Numerous lines of genetic and biological evidence indicate their dual function. The *AVR* genes each co-segregate with the respective avirulence phenotype, have a functional open reading frame (ORF) in avirulent but not virulent isolates, and are expressed by isolates avirulent to the corresponding *R*-gene. The specific recognition of AVRK1 and AVRA10 by MLK1 and MLA10 R-proteins and subsequent cell death was shown in two independent series of experiments by transient expression of *Avrk1* and *Avra10* in barley plants carrying *Mlk1* or *Mla10* resistance genes [4, 5]. Specific recognition of AVRK1 and AVRA10 also induced inaccessibility to subsequent infection and reduced fungal sporulation [4]. Their effector function was demonstrated in two independent experiments. First, when they were transiently overexpressed in susceptible plants lacking the corresponding *R*-gene, they increased the infectivity of *Bgh* [4]. Second, host-induced gene silencing (HIGS) of these genes in susceptible plants caused a reduction in haustorium formation by *Bgh* [5]. Further evidence for effector and AVR functions has been obtained for *Avra10*. This gene is expressed at low levels in conidia that have just landed on the leaf surface, followed by rapid induction within 6 h and high expression until 24 h after inoculation, with a drop thereafter [5]. The interaction between the MLA10 protein and the WRKY2 transcription factor depended on the presence of AVRA10 protein for defense gene activation [6]. An EMS-induced point mutation of *Avra10,* producing premature termination of the coding sequence, rendered the pathogen virulent to plants carrying the *Mla10* resistance gene (i.e. avirulence was lost, so the *Mla10* plant did not detect the fungus and induce effective defences) [4]. Natural *Bgh* isolates virulent to *Mla10* or *Mlk1* do not have functional variants of *Avrk1* or *Avra10* in the corresponding loci*,* in many cases due to the fusion of the *AVR* genes with retrotransposon sequences [4].

*Avrk1* and *Avra10* are homologues (with 64 % nucleotide and 43 % protein identities), and are the first discovered members of the *EKA* gene family (Effectors homologous to *Avrk1* and *Avra10*), with more than 1350 homologues in the *Bgh* genome [7]. The *EKA* family has no known homologues outside powdery mildew fungi [8] but is present in different *formae speciales* of *Blumeria graminis* [9] and in powdery mildew species infecting different host such as pea, grape, plantain or *Arabidopsis thaliana* [9–11]. A previous analysis showed that these *Avrk*1 and *Avra*10 homologues are frequently found in the same ORF as the nucleotide binding (NB) domain of a LINE retrotransposon, that the AVR-homologous and NB domains are expressed in different *Bgh* isolates as a single transcript, and that both type of sequences have coevolved [9].

LINE retrotransposons are almost ubiquitous in fungi, plants and animals [12]. They are able to replicate autonomously, and their mobility is dependent on target-primed reverse transcription [13]. These elements typically consist of an ORF (usually called ORF2) containing a gene encoding a reverse transcriptase (RT) and an endonuclease. In addition, ORF1, usually found in the *L1*, *I* and *Jockey* groups [12, 14], may have been acquired independently on multiple occasions during the evolution of LINE elements [15]. The best studied ORF1 protein (ORF1p) is that in the *L1* superfamily of LINE elements. ORF1p is thought to assist *L1* retrovirus-like particles to gain access to the nucleus, where it can interact with genomic DNA and thus initiate integration through target primed reverse transcription [16]. The ORF1p of the second major superfamily of LINEs (named “*I*”) is only poorly characterized and its function is not yet fully understood [17]. It usually contains at least one non-canonical RNA-recognition motif (RRM) domain and one or more CCHC zinc knuckle motifs [18]. ORF1 proteins evolve much faster than RT proteins, leading to them being very poorly conserved between lineages. That means that RT proteins from distantly related species still show strong homology, while ORF1 proteins have virtually no similarity [18].

In the work reported here, the *EKA* family in the genome of *Bgh* isolate DH14 (BluGen, Blumeria Genome Sequencing Consortium, http://www.blugen.org/, [7]) has been analysed with pipelines from the REPET package, which allows *de novo* detection, classification and annotation of transposable elements (TEs) in whole genomes [19–21]. This method detects TE sequences in the genome and groups them according to a putative common ancestor represented by a consensus sequence (called a TE consensus). The method then identifies the matches between each TE consensus and the genomic sequence (TE fragments) and reconstructs the TE copies in the genome, even if they are nested and degenerated. A TE copy is a chain of matches between each TE consensus and the genomic sequence, each match in the chain being a TE fragment. Hence, a full-length TE copy may correspond to several TE fragments, which, when connected together, correspond to the full TE consensus sequence [19].

Our results show that *Avrk1* and *Avra10* have evolved from part of the ORF1 gene of Class I LINE retrotransposons, which we have named *Kryze* and *Satine*, respectively. The activity of the ancestors of these elements generated a high diversity of degenerate copies. This provides a possible mechanism for the extensive proliferation of the *EKA* family in the *Bgh* genome. These results imply that *Avrk1* and *Avra10* originated from the truncated ORF1 of Class I-LINE retrotransposons, in a recycling and neofunctionalization process in which the retrotransposon genes were recruited by the *Bgh* genome as effectors. The barley plant then evolved to recognize the presence of the *Bgh* parasite through the presence of these retrotransposon-derived effector genes in the fungal genome.

## Results

### *Avrk1* and *Avra10 are* homologues of the ORF1 of LINE 1 retrotransposons

**Fig. 1:**
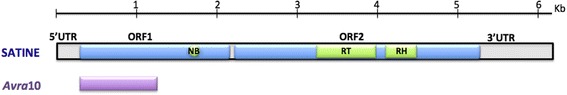
Consensus model *Satine*, which represents a LINE retrotransposon with two ORFs. ORF1 is homologous to *Avra*10 (99 % nucleotide identity of *Avra10* to the corresponding portion of *ORF1*) and contains, in the 3’ region, a sequence coding for a cysteine-rich nucleotide binding domain conserved between different TEs (NB). ORF2 is homologous to the reverse transcriptase and RNase H (RT-RH) of the retrotransposon CgT1 identified in the fungal plant parasite *Glomerella cingulata.* UTR: Untranslated region

Our previous results indicated that the *EKA* gene family was very large and related to TEs. Thus, we based the analysis of the evolution of this family on identification and classification of TE consensus sequences with homology to *Avrk1* and *Avra10*. A first step made use of Bgt_RIX_Inari, a complete TE consensus characterized in *B. graminis* f.sp. *tritici* (*Bgt*) [22] that had been classified as a LINE retrotransposon and contained a region highly similar to *Avra10*. We identified several fragments homologous to Bgt_RIX_Inari in the genome of *Blumeria graminis* f.sp. *hordei* (*Bgh*) isolate DH14 that were then used to build manually a consensus model for *Bgh* that we called *Satine*, which represents a LINE retrotransposon with two ORFs (Fig. 1). ORF1 is homologous to *Avra*10 (99 % nucleotide identity, Additional file 1: Figure S1) and contains, in the 3’ region, a sequence coding for a cysteine-rich NB domain conserved between different TEs [9, 23]. ORF2 is homologous (51 % of amino acid identity) to the reverse transcriptase and RNase H (RT-RH) of the retrotransposon CgT1 identified in the fungal plant parasite *Glomerella cingulata* (anamorph *Colletotrichum gloesporioides*; Fig. 1; [23]).

We then refined the TE annotation of the *Bgh* genome using the TEdenovo pipeline and several rounds of the TEannot pipeline. *Satine* and two previously characterized *Bgh* class I SINE retro-elements (Bgh_EGR1_cons and Bgh_EGH24_cons) [24, 25] were added to the Repbase library [26] used by the TEdenovo pipeline to classify TE consensus sequences. We finally obtained a library of 733 TE consensus sequences (hereafter named Blgr_refTEs). TE annotation with Blgr_refTEs accounted for 67.1 % of *Bgh* genome. Class I LINE and LTR retrotransposons were the most abundant TEs in *Bgh* genome, accounting for 24.5 % and 21.7 % of *Bgh* genome assembly, respectively (Table 1).

**Table 1:** Classification, number, content and genome coverage of TE consensus sequences annotated in *Bgh* genome.

Class, Order	Number of consensus sequences	TE content (%)	Genome coverage (%)
Class I, LTR	358	32.3	21.7
Class I, LINE	241	36.5	24.5
Class I, SINE	20	16.4	11.0
Class II, MITE	6	0.7	0.5
Class II, TIR	7	1.1	0.7
Unclassified	101	13.0	8.7
Total	733	100	67.1

**Fig. 2:**
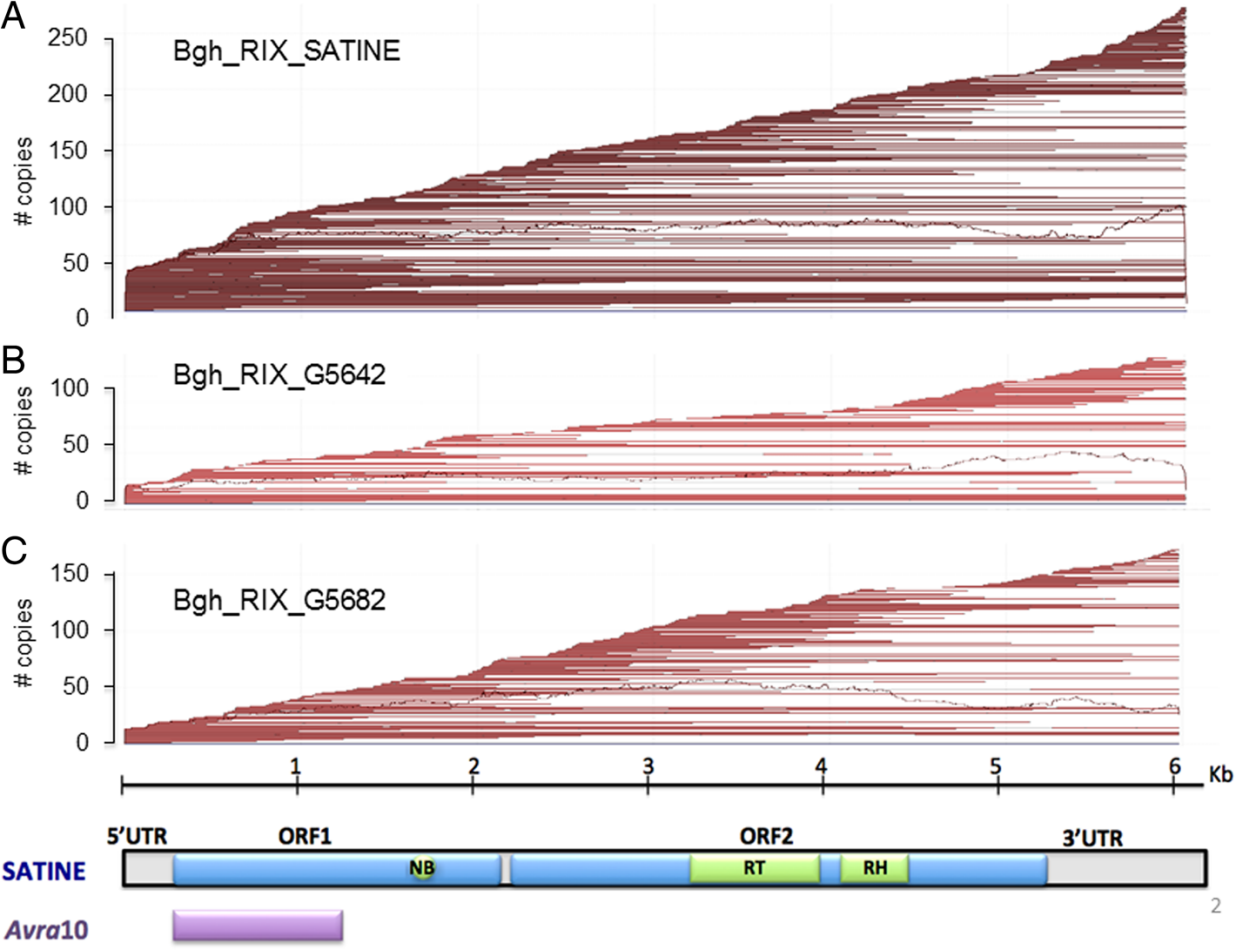
Genome TE copies plotted on the consensus sequences *Satine* (**a**), *Satine*-like Bgh_RIX_G5642 (**b**) and *Satine*-like Bgh_RIX_G5622 (**c**). Brown lines represent the part of the consensus sequence that aligns with each annotated copy. Copies are ordered according to their coordinates (Start, End). The black curve represents the depth of coverage along the reference sequence

In order to identify the TE consensus sequences most similar to *Avra10* and *Avrk1* in the Bgh_refTEs library, we performed a blastx-based sequence comparison [27]. We recovered 13 and 9 consensus sequences highly similar (e-value < 1e-15) to *Avra10* and *Avrk1* respectively and 14 TE consensus sequences similar to both *Avra10* and *Avrk1* (Table 2). These consensus sequences contain 7513 genomic copies and represent 5.68 Mbp (6.5 %) of the *Bgh* annotated genome (88 Mbp). Two TE consensus sequences (Bgh_RIX_G5642 and Bgh_RIX_G5682), classified by REPET as LINE retrotransposons, span the whole *Satine* consensus model (Fig. 2). These three consensus sequences (*Satine*, Bgh_RIX_G5642 and Bgh_RIX_G5682), were used to annotate four, one and two *Satine*-like full-length copies respectively, in the genome. The plot of the location of the genome copies on their respective TE consensus sequences shows that they are smoothly scattered along the whole consensus model, with no break points indicative of chimerization events (Fig. 2). The absence of break points indicates that *Avra10* is part of the original ORF1 of the *Satine* Class I-LINE retrotransposon.

**Table 2:** Metrics of the LINE consensus sequences homologous to AVRK1 or AVRA10

TE	Number of consensus sequences	Consensus max length (bp)	Genome coverage (Mb)	Number of fragments	Number of full length fragments	Number of copies	Number of full length copies
LINE/AVRK1	9	4468	1.43	2370	46	2370	74
LINE/AVRA10	13	6014	1.56	2040	18	1566	37
LINE similar to both AVRK1/AVRA10	14	6083	2.69	3577	39	3577	77
Total	36	6083	5.68	7987	103	7513	188

**Fig. 3:**
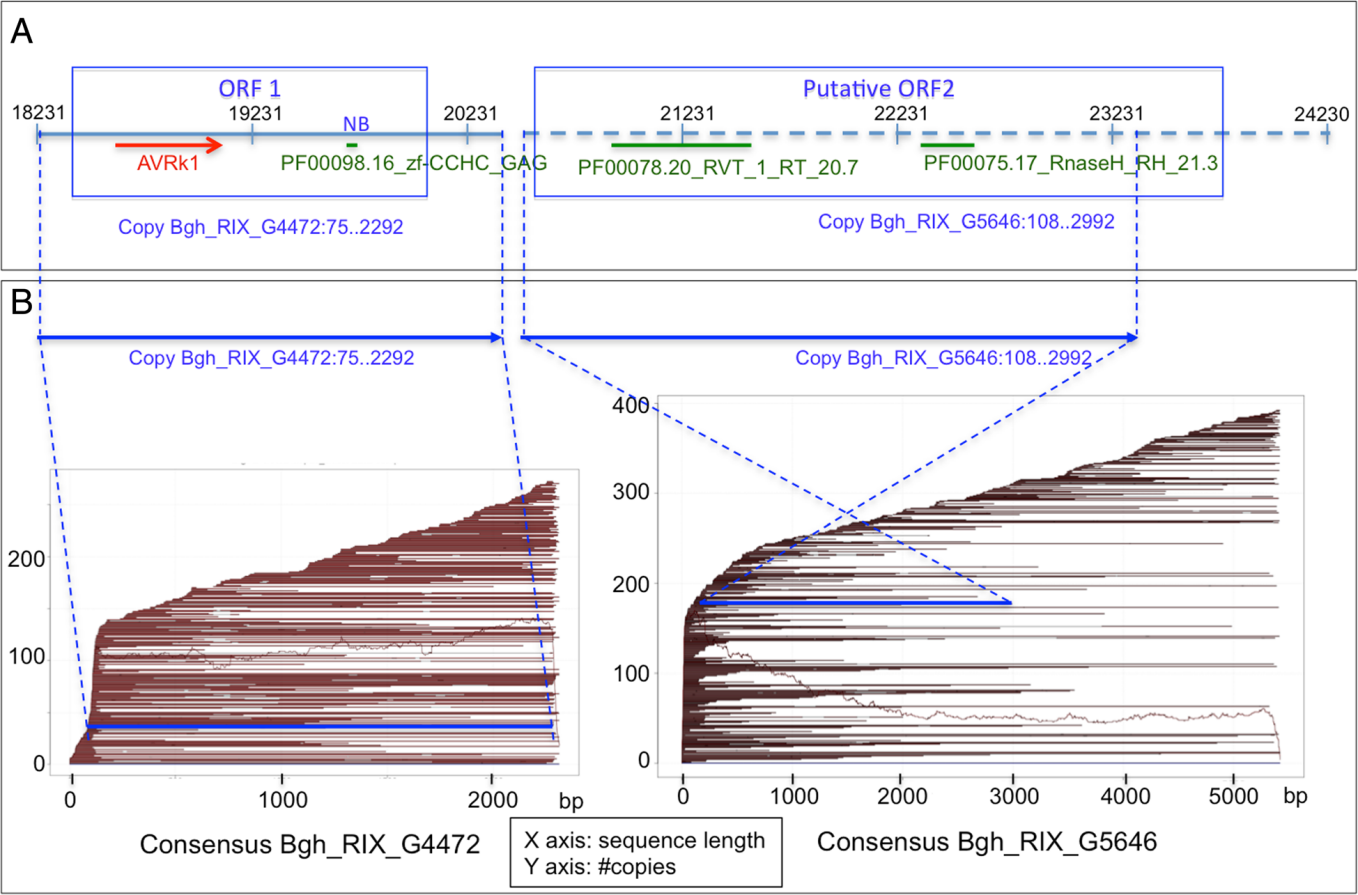
*Kryze* potential genome copy containing an ORF1 homologous to AVRk1 containing a cysteine-rich NB domain (PF00098.16_zf-CCHC_GAG) and a putative ORF2p with RT (PFAM: PF00078.20) and RH (PFAM: PF00075.17) domains. **a** Blgr_v3_contig_001018.fa:18231..24230 bp corresponding to Bgh_RIX_G4472 full-length copy with 3000 bp downstream region. AVRK1 alignment (in red) and domains annotated by PASTEC classifier (green) are represented. **b** Blue lines represent the two TE genomic copies mapped in the context of all the copies (brown lines) plotted to the reference TE consensus sequences Bgh_RIX_G4472 and Bgh_RIX_G5646

Any of the nine TE consensus sequences similar to *Avrk1* could be considered full-length LINE retrotransposons containing both ORF 1 and ORF 2. Seven out these nine TE consensus sequences matched 34 full-length fragments spanning the whole TE consensus sequence. In order to look for putative ORF2 that had been overlooked during TE search and annotation, we extracted the genome sequences of these 34 fragments and searched for LINE-ORF2 domains within the 3000 bp downstream of the ORF1. PASTEC TEclassifier [20] identified the characteristic RT-RH domain at the expected location in one of those sequences (Fig. 3A). Thus, we found a potential genome copy of a full-length LINE retrotransposon with 2 ORFs (that we hereafter name *Kryze*) in a full-length genome copy annotated by TE consensus Bgh_RIX_G4472 (81.5 % of nucleotide identity), extended to 3000 bp downstream. The ORF 1 of *Kryze* is similar to *Avrk1* (67 % amino-acid identity), and the extended 3000 bp containing the ORF2 of *Kryze* are similar (71.5 % nucleotide identity) to the TE consensus Bgh_RIX_G5646 (Fig. 3B).

### Characteristics of the *EKA* family

The alignment of AVRK1 and AVRA10 with the ORF1p of the respective elements (*Kryze* and *Satine*) indicates that both proteins have lost the C-terminal half that contains the conserved NB domain typical of an ORF1p (Figs. 2 and 3). Hence, candidates for other AVR/effector genes may consist of truncated copies of the ORF1p without the region containing the NB domain, thus spanning only the region homologous to AVRK1 or AVRA10. We extracted the 7513 genomic copies homologous to *Avrk1* and *Avra10* and searched for the sequences spanning either the full length or the truncated ORF1p (these last being considered as AVR/effector candidates). We found 135 AVR/effector candidates homologous to AVRK1, 68 AVR/effector candidates homologous to AVRA10, and 90 AVR/effector candidates equidistant from both AVRAK1 and AVRA10 (Table 3). The mean identity among truncated sequences was lower than the identity between the sequences homologous to the full-length ORF1p for all three types of sequences (Table 3). Also, the redundancy was eight times higher in full-length ORF1 sequences than in truncated sequences (3/123 versus 1/294 redundant sequences, Table 3). Hence, truncated sequences are less conserved than full-length ORF1 sequences.

**Table 3:** Number of sequences per group (non-redundant sequences between parenthesis) and mean identity percentage in each group for full length ORF1 and truncated sequences homologous to AVRK1 or AVRA10.

	Full length ORF1 sequences		Truncated sequences before NB domain (putative AVR/effectors)	
	Number of sequences	Mean identity^a^	Number of sequences	Mean identity^a^
AVRK1-like	30 (28)	69.3 %	135 (135)	57.0 %
AVRA10-like	32 (32)	59.9 %	69 (68)	55.2 %
Similar to both AVRK1/AVRA10	61(60)	58.3 %	90 (90)	49.2 %
Total	123 (120)		294 (293)	

^a^Mean identity percentage in each group is based on translated full-length ORF calculated by using the pairwise identity after realigning each pair of sequences and averaging it out for all sequences in the group.

**Fig. 4:**
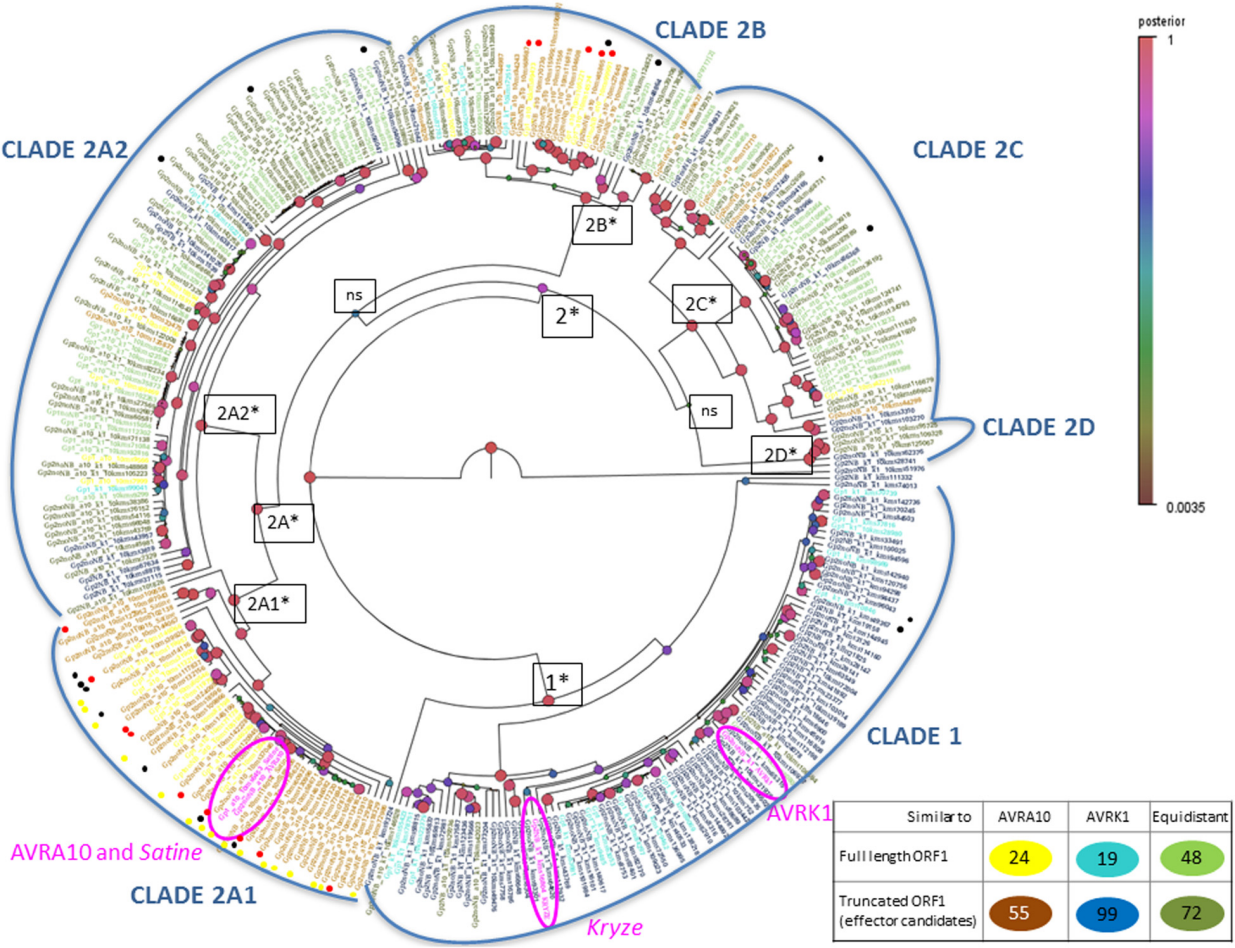
Phylogenetic tree of full length and truncated sequences homologous to AVRK1 or AVRA10 calculated using Bayesian method. Circles on nodes represent posterior probability (*significant nodes with a posterior probability >75 %). Colours represent homology to either AVRK1, AVRA10 or both, and differentiate between sequences corresponding to full length ORF1 or truncated sequences (AVR/effector candidates). Red and black dots indicate proteins expressed in haustoria or/and hyphae, respectively. Yellow dots indicate proteins putatively expressed in haustoria, with one significant peptide and a peptide just below the identity score threshold

**Fig. 5:**
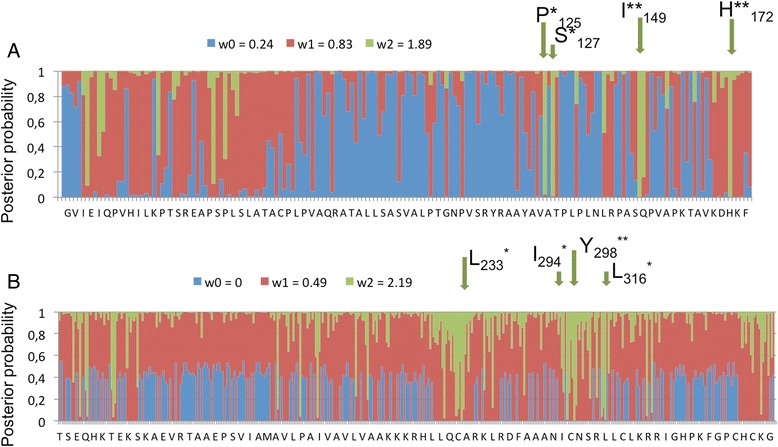
Identification of sites under positive selection of AVRK1 homologues as identified under both M3 (Naive Empirical Bayes, NEB) and M8 (Bayes Empirical Bayes, BEB) models. **a** truncated sequences and **b** full-length sequences. The vertical axis represents posterior probabilities for sites with different *ω* ratios (dN/dS) along the sequence. Positively selected sites (*ω* > 1) are highlighted on top of the graph. *: *p* > 0.95, **:*p* > 0.99 (as reported in the M3 model). w0, w1 and w2 are the three *ω* estimated values in the M3 model

Phylogenetic analysis classifies the sequences from *Bgh* isolate DH14 homologous to AVRK1 or AVRA10 in two clades (Fig. 4). Clade 1 contains mainly AVRK1 homologues and a few equidistant sequences and groups them with the *Kryze* ORF1p and with AVRK1 from isolate CC148. Clade 2 groups all the remaining sequences in five subclades. Subclade 2A1 contains only AVRA10 homologues and groups them with *Satine* ORF1p and with AVRA10 from isolate CC148. The phylogeny indicates that stop codons between the AVR-homologous sequence and the NB domain have appeared several times during the evolution of the *EKA* family, since the truncated sequences appear in the same clades as the full length ORF1 sequences (Fig. 4). We tested if truncated sequences were subject to different selection patterns compared to the full-length sequences, indicative of different functions. We analysed the non-synonymous/synonymous substitution rate ratio (dN/dS = *ω*) to detect positive selection (*ω* > 1) in the full length and truncated sequences homologous to AVRK1 or AVRA10 (Fig. 5). We found evidence of positive selection with variable selective pressure among sites in the four groups of sequences analyzed (*P* < 0.01) (AVRK1-like full length, AVRK1-like truncated, AVRA10-like full length and AVRA10-like truncated). There were different positively selected sites in the AVRK1-like truncated sequences than in the full-length TE ORF1p: sites 125P, 127S, 149I and 172H are positively selected in AVRK1-like truncated sequences and sites 233 L, 294I, 298Y and 316 L, situated after the NB domain, are positively selected in the sequences corresponding to full-length TE ORF1p (Fig. 5). For AVRA10-like sequences, the Likelihood Ratio Test (LRT) did not identify significant positively selected sites either in the truncated sequences or in the full-length TE.

We searched for specific domains in ORF1 predicted proteins in both full length and truncated sequences. There was no similarity with any known ORF1 protein. There was weakly significant similarity (E-value < e^−03^) to retrovirus zinc finger-like domains in both full-length and truncated sequences, whilst predicted coiled coils were only found in full-length ORF1ps.

### EKA protein identification

Several predicted EKA proteins were identified experimentally in infected epidermis (haustoria samples) and in sporulating hyphae from 5–7 dpi infected barley plants (Table 4 and Additional file 2: Table S1). Most peptides contained proline residues, as they were identified from sequences with >8 % proline content and many were detected as hydroxyprolines. The presence of proline is detrimental for mass spectrometry peptide fragmentation using collision induced dissociation (CID) as the “proline effect” leads to a poor fragmentation pattern [28]. This may explain the low Mascot peptide scores, just above the identity threshold, for most detected peptides which contain proline-rich residues. Since proteins could only be identified with two or three peptides, it was not possible to discriminate well between isoforms of closely related proteins, hampering determination of the exact number of proteins in each sample (Additional file 3: Table S2). However, it is clear that several EKA proteins are differentially expressed in various fungal tissues, since most protein hits were identified only in either the haustoria or the hyphae (15 and 14 proteins respectively), with only four proteins identified in both types of sample (Table 4). The possible 33 validated proteins were identified from 15 detected peptides in the haustoria samples and 21 peptides in the hyphae samples. There was almost no overlap of identified peptides between tissues; only the peptide AAAPLPLR was identified in both the haustorial and hyphal samples (Table 4). Most of the identified EKA proteins were AVRA10-like, notably those in haustorial samples (Table 4), with most of the hits in subclade 2A1 that contains *Satine* ORF1p (Fig. 4), including a copy of the TE consensus Bgh_RIX_G5682 that spans the whole *Satine* consensus model. A copy of consensus Bgh_RIX_G5642, which also spans the whole *Satine* consensus model, was found in a hyphal sample. AVRK1-like proteins were only found in hyphal samples (Table 4). Thirteen of the 33 identified proteins are truncated ORF1s and are thus putative AVR/effectors. Most of them (11) are AVRA10-like and appear in haustorial samples, whereas only one truncated protein is AVRK1-like (Table 4). Additional AVRA-10 proteins in the subclade 2A1 were identified in haustoria with one significant peptide and a peptide just below the identity score threshold (Fig. 4 and Additional file 3: Table S2), reinforcing the hypotheses that some haustorium-specific AVRA10 effectors are expressed as proteins.

**Table 4:** Proteomic analysis of haustoria and hyphae samples for experimental identification of EKA proteins.

	Haustoria only	Hyphae only	Haustoria and hyphae
Proteins identified			
AVRK1-like			
Full length ORF1	0	3	0
Truncated (putative AVR/effectors)	0	1	0
Total	0	4	0
AVRA10-like			
Full length ORF1	6	5	1
Truncated (putative AVR/effectors)	8	0	3
Total	14	5	4
Similar to both AVRK1/AVRA10			
Full length ORF1	1	4	0
Truncated (putative AVR/effectors)	0	1	0
Total	1	5	0
Total proteins identified	15	14	4
Total peptides	15	21	1
	ATVPALPR	AGLGRSAGPSITK	AAAPLPLR
	DDRIFLR^a^	AHFHPSTR	
	GLESNIQNMNAIAAALLAKENDVPEVDMVDAEVEK	APKKEAPK	
	KASGPAETSR	ARPSKSGPVK	
	KPTPPTKKSPK	ASDLVALR	
	MLELSQTK	ASIAQFIQAGPGATPPVLPK	
	MPTPPTKQSAK	DQLVTIR	
	MPTPPTKR	EKTIEPAENSTRK	
	NAVSGTAKNR	ELLDSSTSRSVSGIK	
	NMPEVDMVDAEVEKLK	EPPNQPTPVMVSRAPK	
	RATVPALPR	FLPSLPQRQATQGK	
	SPKTATLSSPDNR	KMESDMTER	
	SWAALFPR	KREELLDAAPK	
	TLSPFGGR	LFQMPPTR	
	VPTSFALR	MPTPPSKKLAK	
		QGIPPGSIER	
		SDSRSCKSRPNK	
		SKKSGPVTK	
		SLAHKAAETDK	
		SWAALFPRNASPKPGFR	
		VPTGFTLR	

^a^Peptide DDRIFLR is just below the identity score threshold.

## Discussion

The genomes of powdery mildew fungi are amongst the largest of ascomycete fungi, due to an extraordinary proliferation of TEs [7, 29]. This is probably, in part, the result of the absence of the RIP (Repeat-induced point mutation) pathway to control genetic parasites that is otherwise conserved in all related ascomycetes [7, 30]. TE proliferation and genome expansion may have had deleterious effects on powdery mildews, entailing a considerable loss of genes that has resulted in these pathogens being entirely dependent on living host cells [7]. However, TE activity has also benefited powdery mildews and other eukaryotic pathogens, favoring the expansion and diversification of a broad repertoire of effector genes, as previously shown [31–33]. Here, we show an additional way in which transposition activity contributed to the evolution of *B. graminis*, by the recycling and neofunctionalization of degenerate TE products which may generate new effector genes. The products of at least two of these genes, *Avrk1* and *Avra10* are recognized by the host plant as avirulence proteins and it is conceivable that other members of the *EKA* family may encode avirulence genes.

Our results indicate that the origin of *Avrk1* and *Avra10* is the truncated ORF1 of the retrotransposons *Satine* and *Kryze* respectively (Figs. 2 and 3). *Satine* and *Kryze* are members of the *I* superfamily of LINEs, containing protein domains typical of that superfamily and showing characteristic sequence organization. The *I* superfamily is the only group of LINEs that contains a RNAseH domain in their ORF2, together with the *Randl* group and some members of the *L1* superfamily. Additionally, the *I* superfamily is characterized by having an ORF1 upstream of the ORF2, with zinc finger-like domains [12, 14]. Our structure analysis showed that the ORF1 of *Satine* and *Kryze* contains a zinc finger-like domain characteristic of ORF1 of the LINE I elements. Because ORF1 proteins are known to evolve very rapidly, it is not surprising that no homologues of *Satine* and *Kryze* elements were found outside the powdery mildew fungi [8].

The expansion of *Satine* and *Kryze* lineages may have contributed to the expansion of other protein effectors of the *EKA* family. *Avrk1* and *Avra10* were isolated from *Bgh* isolate CC148, which is avirulent to barley plants carrying resistance genes *Mla10* and *Mlk1* [4]. Isolate DH14 is virulent to barley plants carrying *Mla10* and *Mlk1* and does not contain functional variants of *Avrk1* or *Avra10* in the corresponding loci delimited by genetic and physical mapping [4]. However, this and previous works have found *EKA* homologues in DH14 and other *Bgh* isolates, many of which are transcribed [7–9]. Thus, these homologues may function as effectors in these other isolates and also be *AVR* genes recognized by *R* genes other than *Mla10* and *Mlk1*. We have found 293 truncated copies of *Satine* and *Kryze* ORF1ps that may be AVR/effector candidates because they span the region homologous to *Avrk1* or *Avra10* and lack the downstream sequence containing the NB domain (Table 3). The lower degree of conservation of truncated sequences compared to full-length ORF1 sequences (Table 3) could be due to diversifying selection concomitant with the expansive generation of effectors. Different positively selected sites were found in the AVRK1-like truncated sequences than in the full-length TE ORF1p (Fig. 5), which suggests that the two groups of sequences have different functions. However, the likelihood ratio test did not find significant positively selected sites in AVRA10 homologues. This could be due to a lower number of analyzed *AVRA10* homologues and/or to the fact that AVRA10 homologues are phylogenetically very heterogeneous, in contrast to AVRK1 homologues, which are mostly grouped in a single clade that also contains the *Kryze* ORF1 (Fig. 4). A previous analysis did not find *Avrk1* homologues in *B. graminis* parasitic on oats or ryegrass (*Lolium perenne*), while *Avra10* homologues are present in the genomes of forms of *B. graminis* parasitic on oats, ryegrass, barley and the closely related species rye, wheat and *Elymus repens* [9]. This implies that the *EKA* family proliferated during the evolution of different forms of *B. graminis,* but the *Avrk1* clade (or *Kryze* lineage) only proliferated after the divergence of *B. graminis* on oat and ryegrass from the other forms [34]. Hence, different *EKA* lineages could be related to the adaptation of *B. graminis* to different hosts.

**Fig. 6:**
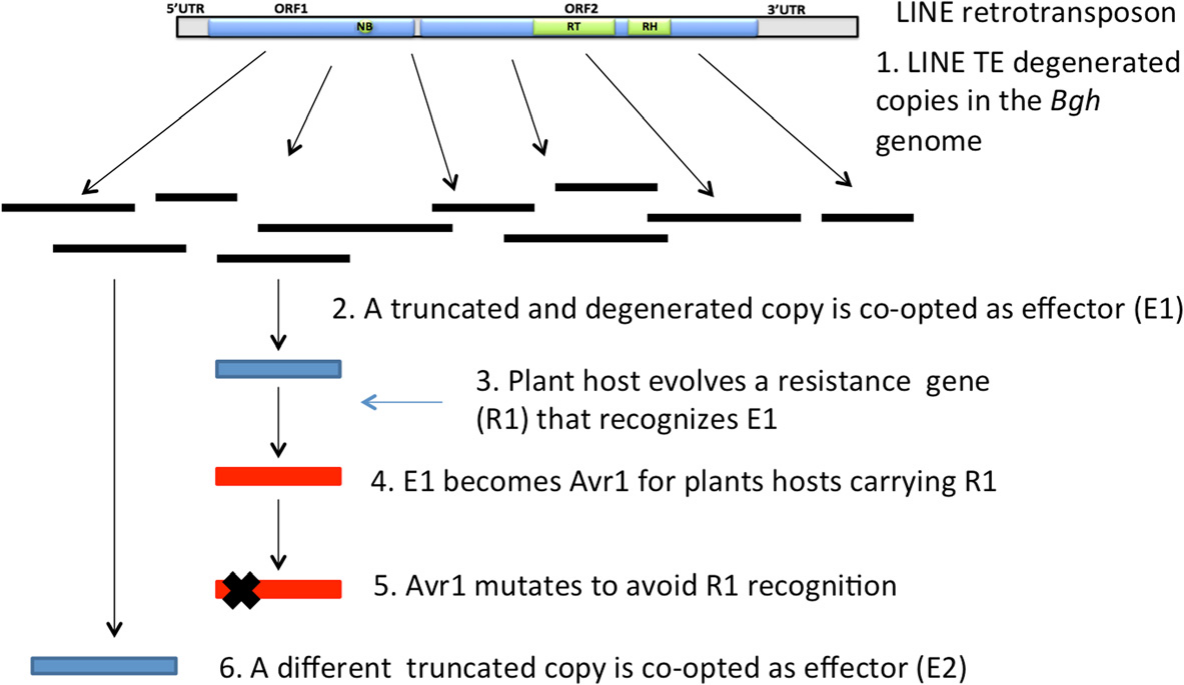
Model for the evolution of the *EKA* family by co-option as effectors by *Bgh* genome. 1. Genome rearrangements after retrotransposon activity produce degenerate copies of LINE retrotransposons in the *Bgh* genome. Some of these copies are truncated ORF1s. 2. One truncated ORF1 is co-opted as effector E1 that enhances pathogenicity. 3. Plant host evolves a resistance gene R1 that recognizes the presence of E1. 4. E1 becomes an avirulence gene (Avr1) if it is recognized by R1 in a gene-for-gene interaction. 5. Avr1 mutates to avoid recognition by R1. 6. *Bgh* co-opts other degenerate copies as effectors that contribute to enhanced pathogenicity if they are not recognized by host *R* genes. During this process, *Bgh* genome and *EKA* copies are evolving, and the copy co-opted at point 6 may not have existed at point 2 and could have come from a different *EKA* ancestor

Our results suggest that there has been a continuous process of recruitment and neofunctionalization of truncated ORF1 copies. Diversification and amplification of this family of effector genes most likely happened as a result of strong natural selection by the host. Loss or mutation of an AVR/effector protein would have resulted in the pathogen not being recognized by the plant host but would have generated selection pressure for its replacement by other effector proteins (Fig. 6). The concept of neofunctionalization and co-option of TE sequences by host genomes was proposed by Brosius and Gould [35], who suggested the term “exaptation” for the phenomenon of “junk” DNA sequences such as TEs acquiring a novel function in the genome. More recently, genome-scale analyses have confirmed that domesticated or exapted TE-derived sequences have contributed diverse and abundant regulatory and protein-coding sequences to host genomes [36]. The new functions which arose from recruitment of TE sequences range from their capture as cis-regulatory sequences at promoter and enhancer regions [37] to the most extensive gene domestication in which single copy genes are well conserved among lineages [38]. Examples of TE domestication are the element of the transib superfamily from which the V(D)J system at the base of the vertebrate immune system evolved [39], the HERV-derived Syncitin genes that play crucial roles in placenta formation [40] and the non-LTR TE origin of telomeres and telomerases [41, 42]. The evolution of the *EKA* family of effectors in a plant pathogenic fungus further indicates the extraordinary diversity of functions which have evolved from retroelements.

AVRK1 and AVRA10 have been shown to function as effectors because they enhance *Bgh* infectivity [4, 5]. It is predicted that *AVR* genes recognized by plant *R* genes should confer a selective advantage if they are to be retained in the pathogen population (reviewed in [43]). While AVRK1 and AVRA10 have effector activities as well as being recognized as avirulence proteins [4–6], their precise functions in the interaction of *Bgh* with its host are unknown. There are several examples that show the transcription of *AVRK1* and *AVRA10* and other members of the *EKA* family [4, 9–11]. Here, we provide the first experimental evidence indicating the production of EKA proteins by *Bgh*. We identified 15 proteins putatively expressed in the haustorial samples and 14 proteins putatively expressed in the hyphal samples (Table 4). This makes a ratio of 1:1 for proteins identified in the haustoria versus the hyphae that, despite the difficulty to determine the exact number of proteins actually expressed, is clearly higher than the ratio found in a global proteomic analysis of these tissues, which is 1:4.5 [44]. Also, 15 and 21 different peptide sequences were identified in haustorial and hyphal samples respectively. Together, these data indicate that the EKA proteins may be more abundant and diverse in haustoria than in sporulating hyphae. This is consistent with the EKA proteins accumulating or being secreted in association with haustorial structures like other fungal effectors [45], although the secretion mechanism is unknown. The identified peptides associated with EKA proteins are almost exclusive to either haustoria or hyphae, with only one peptide shared by both types of samples (Table 4), implying that different EKA proteins may have specific functions in different fungal tissues. These functions may also be specific to the EKA protein subfamily, since most of the haustorium-associated proteins are AVRA10-like but none of the AVRK1-like proteins were found in haustoria. Most of the AVRA10-like haustorium-associated proteins were truncated, unlike AVRK1-like proteins, which were mainly full length and were thus probably functional LINE ORF1s. The functions of the EKA proteins could be related to their biochemical or structural properties. On the biochemical side, they are proline-rich. One example of a fungal proline-rich protein (C1H1) is expressed exclusively in plants and has a role in biotrophy during *Colletotrichum*-host interactions [46, 47]. This gene has orthologs in *C. higginsianum* and *Passalora fulva* (anamorph *Cladosporium fulvum*) that are also proline-rich and are involved in chitin sequestration and camouflage to avoid plant recognition [48, 49]. On the structural side, the ORF1p of superfamily *I* of LINE TEs, from which the *EKA* family derives, contains non-canonical RRM domains through its whole length [18]. AVRK1 and AVRA10 and other EKA members may have conserved part of those domains. Both truncated and full length ORF1 predicted proteins show weak similarities with zinc finger-like domains typical of retroviral Gag-proteins. These domains are involved in protein interactions with RNA, DNA or other proteins [50]. Any of the properties of these domains could have been retained to give a novel function to AVRK1, AVRA10 and other related AVR/effector candidates and thus a selective advantage to *Bgh* individuals carrying them.

Functional innovation by exaptation has been related to extremely stressful situations or crises such as the mass extinction 250 Mya that gave rise to mammals [51]. There are several examples of increased TE activity associated with stress in insects and plants [36]. Indeed, TE mediated genetic variation can be fundamental in the host genome’s evolutionary response to stress, facilitating the adaptation of populations and species to changing environments [52]. The role of TEs in parasite-host coevolution as mutators which increase parasite genomic plasticity has been discussed [32, 53]. Recently, retrotransposons have been shown to increase pathogenesis and virulence by silencing either pathogen avirulence genes or plant defense mechanisms [54, 55]. Here, we provide evidence of a direct role of retrotransposons in virulence as dynamic reservoirs from which new effector genes evolve. It is especially striking that the host plant recognizes the presence of the powdery mildew parasite by the presence of genes which evolved from a genomic parasite – a retrotransposon family – of the fungus.

## Conclusions

Transposable elements account for 67 % of *Bgh* genome size, and Class I-LINE retrotransposons are the most abundant in the *Bgh* genome, representing 24.5 % of genome coverage and 36.5 % of the TE content (Table 1). The sequences homologous to *Avrk1* and *Avra10* (the *EKA* family) within the repetitive DNA landscape of the *Bgh* genome have been identified and analysed, and the origin of these AVR/effector genes has been determined to be the truncated ORF1 of the LINE retrotransposons *Satine* and *Kryze* (Figs. 2 and 3). Thus, *Avrk1* and *Avra10* genes have been co-opted in the *Bgh* genome as effectors from truncated copies of retrotransposons.

DNA repeats of *Satine*-like and *Kryze*-like lineages represent at least 6.5 % of the genome assembly (Table 2). These data indicate a long-standing activity of these two elements in *Bgh* genome, during which a high diversity of degenerate copies has been generated. New effector genes may have evolved from these degenerated copies in a recycling and neofunctionalization process driven by the coevolution between *Bgh* and its host plant (Fig. 6). We have found 293 truncated copies of *Satine* and *Kryze* ORF1s that may be AVR/effector candidates (Table 3) and peptides that indicate translation of these genes (Table 4). These candidates have arisen several times during the evolution of the *EKA* family (Fig. 4), and belong to different lineages that could be related with the adaptation of *B. graminis* to different hosts.

## Methods

### *Satine* consensus manually built

Bgt_RIX_Inari*,* a LINE element detected in *Bgt*-Repeat Library [22], is highly similar to *Avra10*, an AVR/effector gene from *Bgh* isolate CC148 [4]. Homologues of Bgt_RIX_Inari were identified in the genome of *Bgh* isolate DH14 (BluGen, Blumeria Genome Sequencing Consortium, http://www.blugen.org/) with blastn [27] and subsequent manual inspection using dotplot sequence comparison (*DOTTER*, [56]). *Satine* represents the consensus of all the identified *Bgh* copies based on Clustalw [57] alignments.

### TE de novo detection and annotation in *Bgh* genome

The TEdenovo pipeline [19, 20] from the REPET package [https://urgi.versailles.inra.fr/Tools/REPET] was launched on the BluGen *Bgh* assembly of isolate DH14 (BluGen, Blumeria Genome Sequencing Consortium, http://www.blugen.org/) to detect and classify 2251 TE consensus sequences. The TEdenovo pipeline detects TE copies, groups them into families and defines the consensus sequence for each family containing at least three copies. These TE consensus sequences are classified according to their structural features and similarities with known TEs. In addition to Repbase update v16.03 [26] used at the TEdenovo pipeline similarity search step to classify TE consensus sequences, the *Bgt_RepeatLibrary* TE library [22] and a *Bgh*_TE library (unpublished) including *Satine* and two already characterized *Bgh* class I – SINE retro-elements (Bgh_EGR1_cons and Bgh_EGH24_cons) [24, 25] were included. A first TEannot pipeline [21] from the REPET package was launched to annotate TE copies in the genome. A copy is considered as a full-length copy if the assembly of all its fragments represents 95 to 105 % of the TE consensus length. This first TEannot pipeline found full-length copies for at least 1465 consensus sequences (hereafter named Blgr_refTEs_FL library) that were used to launch a second TEannot pipeline. In order to refine TE annotation, Blgr_refTEs_FL was manually curated by curating or deleting potential chimeric consensus sequences and consensus sequences shorter than 0.4 Kb. A library of 733 TE consensus sequences (hereafter named Blgr_refTEs) was finally obtained, including *Satine* and the two already characterized *Bgh* class I – SINE retro-elements Eg-R1_cons and Egh24 [24, 25], and was used to annotate the genome TE content. The TE consensus sequences (Fasta) and Annotation file (GFF) are available at: https://urgi.versailles.inra.fr/download/fungi/TEs/ (http://doi.org/10.15454/1.4454357532232493E12 and http://doi.org/10.15454/1.44543859671938E12).

### Search for the full-length LINE retrotransposon containing *Avrk1* in ORF1

Using Blgr_refTEs_FL genome annotation, we extracted all the full-length fragments (alignment length between 95 and 105 % of the TE consensus length) corresponding to the seven TE consensus sequences that were homologous to full-length *Avrk1* from *Bgh* isolate CC148 [4] at the expected location of an ORF1 of a LINE retrotransposon, together with 3000 bp flanking sequence on each side. We obtained 34 extended TE genome fragments that we annotated using PASTEC TEclassifier [20].

### Search for *EKA* TE families

We searched for *EKA* TE families using AVRA10 (gi|111035036|gb|DQ679913| *Blumeria graminis* f. sp. hordei isolate CC148 *Avra10* mRNA, complete CDS) and AVRK1 (gi|111035034|gb|DQ679912.1| *Blumeria graminis* f. sp. hordei isolate CC148 complete CDS) protein sequences as query for a blastx search [27] (e-value threshold 1e-05) in the Blgr_refTEs TE consensus library. We divided the results into three categories: two containing TE consensus sequences very similar to and AVRK1 and AVRA10 respectively (e-value < 1e-15), and a third containing TE consensus sequences equidistant from AVRK1 and AVRA10*.*

### Plot *Satine*-like and *Kryze*-like copies on TE consensus

We used PlotCovergage.py program from the S-MART package [58] to plot the coordinates of *Satine*-like and *Kryze*-like copies on their respective TE consensus sequences.

### Search for AVR/effector candidates

We used blast (tblastn) [27] to search AVRK1 and AVRA10 peptide sequences against in-house databases of copies of TE consensus sequences similar to AVRK1 and AVRA10 (e < = 1e-05, length of hit > 50 amino acids). We discarded the shorter sequences (length < 75 % of AVRK1/AVRA10 length) and aligned the remaining sequences and AVRK1 or AVRA10 using muscle [59], identified the 63 amino acids that represent the AVRK1/AVRA10 core sequence [4] and discarded the aligned sequences with a shorter core sequence (<75 % AVRK1/AVRA10 core). We also discarded the sequences with a stop codon in the region aligned with AVRK1 or AVRA10. The resulting sequences were used to compile a database of predicted 415 EKA proteins (Table 3). We identified the NB domain in the remaining sequences using fuzzpro [60] and classified the sequences as full-length or truncated depending on the position of the stop codons.

### Phylogenetic analyses

We combined the protein sequences from both AVRK1 and AVRA10 analyses, aligned them with muscle [59] and edited the alignment using jalview [61]. We selected the sequences that were at least 90 % of the length of AVRK1 and removed 27 sequences that were redundant in that region, resulting in 317 sequences for the phylogenetic analyses. We used the BEAUti/BEAST software package v. 1.8.1 [62] to infer Bayesian phylogeny (the subsequent cited programs are also distributed with BEAST). We carried out two independent runs of 100,000,000 generations with sampling every 1000 generations. The runs were combined using Tracer v. 1.5.0. The Effective Sample Size (ESS) for the posterior distribution, likelihood and treeLikelihood showed satisfactory values, showing that the MCMC chain has been run for long enough to get a valid estimate of the parameter. The trees from both runs were combined using LogCombiner. In order to limit the analysis to the part of the trace that is in equilibrium, we discarded the initial 10,000,000 runs (burn-in value equal to 10 %). We generated a summary tree using TreeAnnotator and visualized it with Figtree. The sequence alignment used to produce the phylogeny is available at LabArchives. http://doi.org/10.6070/H4X34VG0.

### Positive selection analysis

We used the program codeml from the package PAML 4.8 [63] to detect positive selection that may affect some sites in either AVRK1-like or AVRA10-like group of sequences. We used six different codon-based likelihood models (M0, M1a, M2a, M3, M7 and M8) to detect positively selected amino acid sites. The models are implemented in a maximum likelihood framework and tested using a likelihood ratio test (LRT).

### Search for protein structural domains

We used Batch Web CD-search tool [64], CDART tool [65], HMM Superfamily search [66] and HHpred [67] to look for known structural domains.

### EKA protein identification

The database of 415 predicted EKA proteins including AVRK1 and AVRA10 (Table 3) was merged with the *Bgh* DH14 protein database on the Blugen website (http://www.blugen.org/) in order to have a database search space that better reflects a complete proteome. The resulting database (of 6,885 sequences and 3,289,563 amino-acid residues) was used to query the mass spectrometry (MS) data sets from a large-scale analysis of the haustorial and hyphal proteomes of *Bgh* DH14 [44]. The original MS and Mascot data as well as associated metadata are publicly available and can be retrieved from the PRIDE database (Accession Numbers 15917–15924; http://www.ebi.ac.uk/pride/). The search parameters were the same as in [44], but using Mascot vs. 2.4 (MatrixScience, London) as search engine. A first survey Mascot search was performed by including a “tolerant search” to allow any post-transcriptional modifications to be detected. From this search it was seen that most of the peptides identified were rich in proline (P) and many were detected as hydroxyprolines. A subsequent search was then performed adding oxidised P in the variable modification. Proteins were validated by the identification of at least two significant peptides, with a Mascot score above 20 and above the identity threshold (*p* < 0.05).

### Availability of supporting data

The data sets supporting the results of this article are available in:

INRA. http://doi.org/10.15454/1.4454357532232493E12. TE consensus library generated from *Bgh* genome assembly.

INRA. http://doi.org/10.15454/1.44543859671938E12. *Bgh* genome: TEs annotation file.

LabArchives. http://doi.org/10.6070/H4X34VG0. Sequence alignment used to produce the phylogeny.

PRIDE database (Accession Numbers 15917–15924; http://www.ebi.ac.uk/pride/). Original MS and Mascot data and associated metadata for protein identification.

## Additional files

Additional file 1: Figure S1. Alignment of nucleotide sequences of *Avra10* gene and *Satine* ORF1. (PDF 131 kb) Additional file 2: Table S1. Mass spectometric data results for proteomic analysis using the database of predicted EKA proteins merged with the *Bgh* DH14 protein database. (XLSX 33 kb) Additional file 3: Table S2. Peptides present en each protein accession. (XLSX 21 kb)

## Abbreviations

AVR: Avirulence
*Bgh*: *Blumeria graminis* f.sp. *hordei*
*Bgt*: *B. graminis* f.sp. *tritici*
BluGen: Blumeria Genome Sequencing Consortium
CID: Collision induced dissociation
*EKA*: Effectors homologous to *Avr**k**1* and *Avr**a**10*
ESS: Effective Sample Size
ETI: Effector-triggered immunity
HIGS: Host-induced gene silencing
LRT: Likelihood Ratio Test
MS: Mass spectrometry
NB: Nucleotide binding
ORF: Open reading frame
ORF1p: ORF1 protein
R: Resistance
RIP: Repeat-induced point mutation
RH: RNase H
RRM: RNA-recognition motif
RT: Reverse transcriptase
TE: Transposable Element
