# Supplementary material for: Evolution of the EKA family of powdery mildew avirulence-effector genes from the ORF 1 of a LINE retrotransposon
Source: BMC Genomics. 2015 Nov 10;16:917. doi: 10.1186/s12864-015-2185-x (PMC4641428; doi:10.1186/s12864-015-2185-x)
Supplement: Additional file 1: Figure S1. — Alignment of nucleotide sequences of Avra10 gene and Satine ORF1. (PDF 131 kb) [file 12864_2015_2185_MOESM1_ESM.pdf]

```
AVRa10  ............................................................
Satine  AATTCGAGAAGCCCCCAATTTGCCCATCCCACCCTTTTATCCATCTCACCCTTTGAGGAA

AVRa10  ............................................................
Satine  CGAATCGAATCGAAATTGTTTGCCATCGGCGGCATTTCTAAAATGCCTCCGACGCGTAAA

                                                                1
AVRa10  ........................................................ATGG
Satine  ACGCAAAAAAACGCGATCAGCGGAATCACCAAAGATCGCGTCATCAAACCCTACCAATGG

                  10        20        30        40        50        60
AVRa10  ACTCCAGCCATTCAAAACCACGCGAGAAACATGCTAACAAGAAAAGGACTCGAGTCCAAT
Satine  ACTCCAGCCATTCAAAACCACGCGAGAAACATGCTGACAAGAAAAGGACTCGAGTCCAAT

                  70        80        90       100       110       120
AVRa10  CTTCAAAATATGAACACCATCGCGGCTGCGCTCCAAGCGAAGGAGAACGATGTCCCCGAG
Satine  CTTCAAAATATGAACACCATCGCGGCTGCGCTCCAAGCGAAGGAGAACGATGTCCCCGAG

                 130       140       150       160       170       180
AVRa10  GTAGATATGGTGGACGCAGAAGTCGAGAAGCTAAAAACCCTCGCCGATTCAATTTTGGCA
Satine  GTAGATATGGTGGACGCAGAAGTCGAGAAGCTGAAAACCCTCGCCGATTCAGTTTGGGCA

                 190       200       210       220       230       240
AVRa10  TCCTCCAGCCCAGAAGATGCAGAGCAAGTCTTGGATCCCACCGGATCCCCCA.AAAAACC
Satine  TCCTCCAGCCCAGAAGATGCAGAGCAAGTCTTGGATCCCACCGGATCCCCCAGAAAAACC

                  250       260       270       280       290       300
AVRa10  GTCGTCGCACCCGAGAAGCGCGCTGCGGTTCCAGCACTACCGCGACCAGTAGAAGTGAGG
Satine  GTCGTCGCACCCGAGAAGGGCGCTACGGTTCCAGCACTACCGCGACCGGTAGAAGTGAGG

                  310       320       330       340       350       360
AVRa10  CCCACGAAAGAGGCCGACATACCCACAGCCAAGGCAGCAGGAGCCGAAAACTCTTCGCTG
Satine  CCCACGAAAGAGGCCGACATACCCACAGCCAAGGCAGCAGGAGCCGAAAACTCTTCGCTG

                  370       380       390       400       410       420
AVRa10  GACCAACCGCCTACAGCACCAGAAGCAGCAGGCAGAGAAAAAATATTCCCCCCAGAATTG
Satine  GACCAACCGCCTACAGCACCAGAAGCAGCAGGCAGAGAAAAAATATTCCCCCCAGAATTA

                  430       440       450       460       470       480
AVRa10  CTGGCAGCGATCGAAGCGGAGGAGCGCAGGGCAAGGCAAAAAGCTGCGCAATTCACAATA
Satine  CTGGCAGCGATCGAAGCGGAGGAGCGCAGGGCAAGGCAAAAAGCTGCGCAATTCACAATA

                  490       500       510       520       530       540
AVRa10  TGCTCGACGGCTATTAGCAGTGTCGAGGCAGCCCTTCAACCGCTCTCCACCGGTGAAGAC
Satine  TGCTCGACGGCTATTAGCAGTGTCGAGGCAGCTCTTCAACCGCTCTCCACCGGTGAAGAC

                  550       560       570       580       590       600
AVRa10  AAAAATTTTGTAGACTCCATCAAAGTCTACTTGCGAGCAGCAATCGCGCAATTCGTGGCG
Satine  AAAAATTTTGTAGACTCCATCAAAGTCTACTTGCGAGCAGCAATCGCGCAATTCGTGGCG

                  610       620       630       640       650       660
AVRa10  GCCGGTCCGAGTACCGCTCCGCCAGTCCTACCCCAACGGCCAAGTGTCGGGA...CCCCT
Satine  GCCGGGCCGAGCACCGCTCCGCCAGTCCTGCCTAAACGACCAATTGTCGCAACTCCCCTC

                  670       680       690       700       710       720
AVRa10  GCGACCGTAATCCCAGTAGCCATTCCTCGGAATATTTCAAAGGCTACTGCACCTCTCCCG
Satine  GCGACCGCCATCCCAGTAGCTGTTCCTCGGAATATTTCAAAGGCTGCAGCACCTCTCCCG

                  730       740       750       760       770       780
AVRa10  CTCAGAAGCACCTGGGCCACTGTCACCCGAGCAGGACACCAAAAGTCAGGCGCACAAGCT
Satine  CTCAGAAGCACCTGGGCTACTGTCACCAGAGCAGGACACCAAAAGTCAGGCGCACAAGCC

                  790       800       810       820       830       840
AVRa10  CCTGCAAATATCACCTCGGTTTCGGCTGCCCCATCTGCCAGGAAACAAGAGAAGAAGCCG
Satine  CCTGCAAACATCATCTCGGTTTCGGCTGCCCCATCTGCCAGGAAACCAGAGAAGAAGCCG

                  850
AVRa10  GCAGCATCAACAACAGCA..........................................
Satine  GCAGCATCAACAACAGCATCGAAAGATGACCGAATCTTTCTGCGTCTTGATGCAAACCAC

AVRa10  ............................................................
Satine  GAGTGGAGACAGCTCTCTCCAGCTGGCGTCCGCGAAGCCGTCGCTAAACAAACAAATTGC

AVRa10  ............................................................
Satine  ACCCCTGCTGATGTCGACCAGGTGCAACGCGTTCCCACTGGCTTTGCCATCCGTGCCAAA

AVRa10  ............................................................
Satine  AACCCGGATGCAAAAACTCGACTGCTGGAGGCCTCCAGCACCTTCACACAAGTGGAGGCA

AVRa10  ............................................................
Satine  AAACTAGAACCACAAAGTGATCTAGTTTCCCTCCGGATCGCAACGGTTCCAGTCGCCGTG

AVRa10  ............................................................
Satine  TTCAGCTTAGAAGGACGAGTCGAAGTCACTGCCGAAATGGTGGCCGCCGAAATCTTTAGA

AVRa10  ............................................................
Satine  GTAACCAGTTGCACTCCAGACCGAGTAAGGATACACGGAAAAACTAAGCTCGGCGCTCCC

AVRa10  ............................................................
Satine  CACAGATCCTGGTCGGCCTTGTTTCCCAGGAACGCTAGCCCAAAGCCTGGATTCCGACTC

AVRa10  ............................................................
Satine  TTCGACGACTCAGGAATAGCAACCATCCAACGAAACCGCCCTCCCATACAGCAATGTAGG

AVRa10  ............................................................
Satine  CGATGTCTCGGTTTCCACGCCACCCGTGGGTGTTCTCGCGCGCCAGCCTGCTGGAACTGC

AVRa10  ..........................................
Satine  GGATCCACCATGCACTCGGCATTCGAGTGTAAAGCAGCCCTC
```
